# Green evaluation of human plasma levels of metformin, linagliptin, and empagliflozin using HPLC and HPTLC methods: a pharmacokinetic study

**DOI:** 10.1186/s13065-026-01726-z

**Published:** 2026-02-03

**Authors:** Osama I. Abdel Sattar, Hamed H. M. Abuseada, Mohamed Saleh Emara, Islam Selim, Mohamed A. Ali

**Affiliations:** 1 Pharmaceutical Analytical Chemistry Dept Faculty of Pharmacy, Al-Azhar University, Nasr City, Cairo, 11751 Egypt; 2 Pharmaceutical Analytical Chemistry Department Faculty of Pharmacy, Badr University in Cairo (BUC), Badr city, Cairo, 8829 Egypt; 3 Faculty of Biotechnology, Badr University in Cairo (BUC), Badr city, Cairo, 8829 Egypt

**Keywords:** Plasma, Pharmacokinetic, HPLC, Empagliflozin, Linagliptin, Metformin

## Abstract

Two simple, rapid, cost-effective, and environmentally friendly chromatographic methods were developed and validated for the simultaneous determination of metformin (MEF), linagliptin (LIN), and empagliflozin (EMP) in human plasma, with successful application to pharmacokinetic study. Plasma sample preparation was performed using a straightforward protein precipitation technique employing acetonitrile: methanol: trichloroacetic acid (50:49:1, by volume), which provided high extraction recovery and minimal matrix interference. The first method was based on high-performance liquid chromatography with diode array detection (HPLC–DAD) using an ODS Hypersil C18 column and isocratic elution with a mobile phase consisting of acetonitrile, methanol, and phosphate buffer (pH 3) in a ratio of (40:40:20, by volume), at a flow rate of 1.3 mL/min, with detection at 230 nm. The second method employed high-performance thin-layer chromatography (HPTLC) with densitometric detection at 225 nm, using silica gel 60 F254 plates and n-hexane: methanol: glacial acetic acid (6:3:1, by volume) as the developing system. Excellent linearity was achieved over concentration ranges of 85–1650 ng/mL for MEF, 50–1100 ng/mL for EMP, and 45–950 ng/mL for LIN using the HPLC method, and 500–2800, 100–800, and 50–550 ng/band, respectively, using the HPTLC method, with correlation coefficients exceeding 0.998. The lower limits of quantitation for the HPLC method were 85, 50, and 45 ng/mL for MEF, EMP, and LIN, respectively. Both methods demonstrated satisfactory accuracy, precision, recovery (> 92%), stability, and negligible matrix effects in accordance with European Medicines Agency guidelines. The validated methods were successfully applied to a pharmacokinetic study in healthy volunteers, yielding mean Cmax values of 877.5 ± 162.2 ng/mL (MEF), 576 ± 87.5 ng/mL (EMP), and 680.8 ± 7.9 ng/mL (LIN), with Tmax values of 2.42 ± 0.38, 1.5 ± 0.61, and 5.3 ± 0.52 h, respectively. The obtained pharmacokinetic parameters were consistent with reported literature, confirming the reliability and clinical applicability of the proposed green bioanalytical methods.

## Introduction

There is no indication that the prevalence of diabetes mellitus is going to decline globally. According to World Health Organization (WHO) data, the prevalence of diabetes increased by 80% between 1980 and 2014 [1]. This increase adds a burden of excess morbidity, mortality, and health care expenses, and it disproportionately impacts low- and middle-income nations in comparison to high income countries (HIC) [2]. In particular, the prevalence of diabetes was 12.2% in the Middle East and North Africa in 2019 and is predicted to rise by 96% between 2019 and 2045, second only to the African region, which is predicted to have a 143% increase [3]. In contrast, the prevalence is predicted to rise by 15% and 33%, respectively, in Europe and North America/Caribbean regions over the same period. Furthermore, throughout the Middle East and North Africa, 44.7% of individuals with type 2 diabetes (T2DM) are not aware that they have the disease [3]. Therefore, quantitative analysis of the most popular and recently used medications for the treatment of type 2 diabetes]specifically, metformin (MEF), linagliptin (LIN), and empagliflozin (EMP)] in Egypt and the Middle East and North Africa region requires quick, verified, sensitive, and selective analytical techniques.

**Fig. 1:**
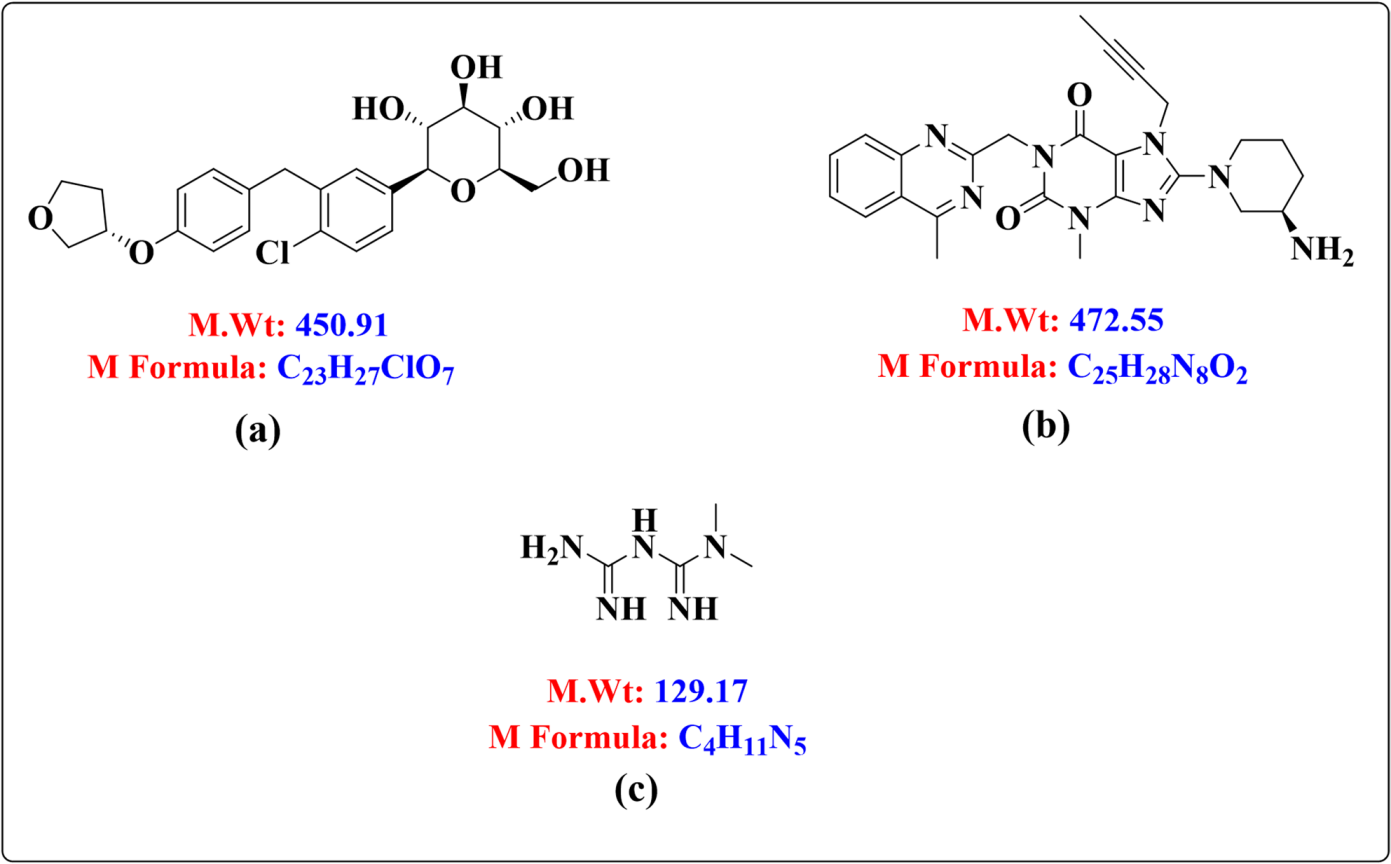
Chemical structures of (**a**) EMP, **b** LIN, and **c** MEF

The innovative technique of treating type 2 diabetes with EMP, a selective sodium-glucose co-transporter 2 inhibitor (SGLT2i), involves increasing the excretion of glucose in the urine [4]. The chemical formula is (2*S*,3*R*,4*R*,5*S*,6*R*)−2-[4-chloro-3-[[4-[(3*S*)-oxolan-3-yl]oxyphenyl]methyl]phenyl])6-(hydroxymethyl)oxane-3,4,5-triol. It has a molecular weight of 450.91 g/mole, and the molecular formula is C_23_H_27_ClO_7_ (Fig. 1a). It is a white to yellowish powder that is very slightly soluble in water. It is also slightly soluble in acetonitrile and ethanol, sparingly soluble in methanol, and practically insoluble in toluene.

The determination of EMP in different matrices has been reported using a variety of analytical techniques, including HPLC [5] and LC-MS/MS [6].

LIN is a dipeptidyl peptidase IV (DPP-IV) inhibitor. The USFDA has approved linagliptin as a monotherapy or in combination with other commonly prescribed oral hypoglycemics for T2DM [7]. Chemically, it is “(*R*)−8-(3-amino-piperidin-1- yl)−7-(but-2-ynyl)−3-methyl-1-(4-methyl-quinazolin-2-ylmethyl)−3,7-dihydro-purine-2,6-dione” as shown in (Fig. 1b). LIN is a white to yellow crystalline solid with a molecular formula of C_25_H_28_N_8_O_2_. It has a molecular weight of approximately 472.5 g/mol. LIN is very slightly soluble in water (0.9 mg/mL). It is soluble in methanol (about 60 mg/mL), sparingly soluble in ethanol (about 10 mg/mL), and very slightly soluble in isopropanol and acetone. Its melting point is reported to be between 190 and 196 °C.

Several analytical techniques, including LC-MS/MS [6], HPLC [8–13], and HPTLC [14, 15] have been published for LIN determination in diverse matrices.

One of the biguanide class of medications, MEF is frequently used in conjunction with other oral hypoglycemic medications to treat type II diabetes. It inhibits hepatic gluconeogenesis and glycogenolysis. N, N-dimethylimidodicarboimide diamide is its chemical formula (Fig. 1c). MEF is a white, crystalline powder that is freely soluble in water and slightly soluble in alcohol. It has a bitter taste and is practically insoluble in acetone, ether, and chloroform. Its molecular weight is 165.63. It has a pKa of 12.4, making it a strong base, and a pH of 6.68 in a 1% aqueous solution.

Several analytical methods have been reported for the determination of MEF, as it has a wide range of therapeutic uses and combined multi-drug therapy for a variety of diseases. These methods including: UV spectrophotometric method [16–18], chemometric methods [19, 20], electrochemical methods [21, 22], GC–MS/MS methods [23], HPTLC methods [24–26], HPLC methods [27, 28], LC-MS/MS method [29].

This study presents two straightforward, rapid, and validated chromatographic methods, high-performance liquid chromatography (HPLC) and high-performance thin-layer chromatography (HPTLC), for accurate bioanalysis of metformin, linagliptin, and empagliflozin in human plasma with pharmacokinetic applications.

The objective of this research is to provide chromatographic methods that are easy to use, quick, accurate, and verified for the simultaneous measurement of MEF, LIN, and EMP in human plasma. Demonstrating their rationality for the stated purpose and potential utility for pharmacokinetic research was another primary goal of the current bioanalytical process. The methods for bioanalytical method validation were verified in accordance with the European Medicines Agency’s (EMA) recommendations [30].

The novelty of the current study lies in developing and validating two cost-effective, rapid, and reliable chromatographic methods (HPLC-DAD and HPTLC-densitometry) directly applicable to human plasma, with successful implementation in a pharmacokinetic study. In contrast to earlier work, the present methods employ a simple protein-precipitation extraction with minimal solvent volumes, reducing both analysis time and environmental impact. The proposed methods provide a greener, cost-effective, and faster alternative with simple protein precipitation and minimal solvent use, thus filling an important analytical gap.

Furthermore, their degree of greenness and sustainability has been prospectively evaluated in comparison with reported methods using modern green assessment tools such as the more comprehensive MOGABI approach [31], which considers solvent safety, energy efficiency, waste generation, and overall applicability. Thus, the proposed methods combine clinical applicability with improved eco-friendliness, offering a sustainable alternative to previously reported analytical procedures.

## Experimental

### Instruments

The protein precipitation extraction process was carried out using a Bio-Base centrifuge type BTBK-12HRT3 (China). The HPLC-DAD method (Thermo ScientificTM, Waltham, Massachusetts, US) was performed using an Agilent 1200 Diode Array Detector (DAD) detector, a G1311A quaternary pump, and a G1329B auto-sampler. The HPLC system model 1200 (Agilent, USA) had a chromatographic column of ODS Hypersil C18 (250 × 4.6 mm, 5 μm particle size). The TLC-Densitometric method was performed using TLC aluminum plates (20 × 10 cm, 0.20 mm) that had been previously coated with silica gel 60 F254 (EMD Millipore, Sigma Aldrich). An automated applicator, the CAMAG LINOMAT V (Muttenz, Switzerland), fitted with a 100 µL microsyringe from Hamilton, Switzerland, was used to apply the samples. The plates were scanned at 225 nm using TLC Scanner 3, which was powered by winCATS software.

## Materials

### Pure sample

Pure EMP (99.78%, Batch No: 03470), LIN (99.91%, Batch No: 9854), and MEF (99.98%, Batch No: 2506208) were kindly supplied by Eva pharma Company (Egypt), their purity was according to the certificate of analysis from the company.

### Chemical and reagents

Acetonitrile HPLC grade (Honeywell, Germany) is one of the analytical grade chemicals and reagents that were employed throughout the work. Methanol HPLC quality (Germany, Honeywell). Water HPLC quality (Germany: LiChrosolv). Acetic acid tri-chloro (PIOCHEM, Cairo, Egypt). N-hexane (PIOCHEM, Egypt’s Cairo). Acid from glacial ice (PIOCHEM, Cairo, Egypt).

### Standard solutions

EMP, LIN, and MEF stock solutions containing 100 µg/mL were produced individually in methanol. The equivalent stock solutions for EMP, LIN, and MEF were serially diluted using the same solvent to create the working solutions. When not in use, all solutions were kept refrigerated between 2 and 8 °C.

## Method development

### Samples collection and storage

In K2-EDTA vacationer collection tubes (BD, Franklin, NJ, USA), blood samples] were collected in a fasted state (at least 10 h of fasting prior to sampling) from the healthy volunteers [were obtained after oral administration of metformin (850 mg), linagliptin (5 mg), and empagliflozin (10 mg) at pre-dose and at 0.5, 1, 1.5, 2, 3, 4, 8, 12, 16, 24 and 48 h. The tubes were centrifuged for 10 min at 4000 rpm to extract the plasma. Before being used, the obtained plasma samples were kept in storage at −80 °C.

## Procedures

### Sample preparation and extraction procedure (protein precipitation technique)

500 µL of a mixture solution of acetonitrile: methanol: trichloroacetic acid, (50: 49: 1 by volume) was added to 500 µL of each spiked calibration plasma standard or QC sample using a protein precipitation and extraction procedure. The solutions were vortexed for one minute following 10 min of centrifugation at 4000 rpm. The supernatant was transferred to a glass vial for HPLC-DAD analysis and to a microsyringe for HPTLC examination.

### HPLC-DAD method

#### Chromatographic conditions

 The ideal wavelength for simultaneous detection of the target analytes was determined to be 230 nm, and the DAD detector was adjusted to operate within the range of 210–350 nm. An ODS Hypersil C18 column (250 × 4.6 mm, 5 μm particle size) was used for the chromatographic separation. A mobile phase consisting of an acetonitrile: methanol: phosphate buffer (pH 3) mixture of (40: 40: 20; by volume) was isocratically eluted at a flow rate of 1.3 mL/min. The injection volume was 10 µL.

#### Construction of calibration graphs (Linearity)

A number of MEF, EMP, and LIN calibration standards were produced to verify the linearity. The calibration standards for LIN were 45, 90, 180, 400, 700, and 950 ng/mL, and for MEF they were 85, 170, 340, 680, 1200, and 1650 ng/mL; for EMP they were 50, 100, 200, 400, 800, and 1100 ng/mL. They were made by mixing 450 µL of human plasma free of drugs with 50 µL of each drug’s corresponding working solution, then analyzing the mixture. The peak areas of the medications under study were plotted against the drug’s concentration to create the calibration curves for MEF, EMP, and LIN. Throughout the entire investigation, the concentration of each drug was determined using the obtained regression equations.

### HPTLC-densitometric method

#### Chromatographic conditions

Using a Camag Linomat V automatic applicator and a 100 µL Hamilton micro-syringe, samples were applied as bands to TLC aluminum plates (20 × 10 cm, 0.20 mm) that had previously been coated with silica gel 60 F254. The bandwidth was adjusted by 6 mm. Each band was spaced 1 cm apart and 1.5 cm from the lower border of the plate. For twenty minutes, the mobile phase was pre-saturated in the chromatographic chamber. The plates were developed to a distance of around 8 cm using an ascending technique and a mobile phase consisting of n-hexane: methanol: glacial acetic acid (6:3:1; by volume). The plates were allowed to air dry at ambient temperature before being scanned at 225 nm using the Camag TLC scanner III. When this TLC scanner was in absorbance mode, the radiation source was a deuterium lamp. A scanning speed of 20 mm/s was employed, and the slit size was kept at 3 mm ×0.45 mm.

#### Construction of calibration graphs (Linearity)

To establish the linearity, a series of calibration standards of MEF, EMP, and LIN were prepared. The calibration standards of MEF were from 500 to 2800 ng/band, 100 to 800 ng/band for EMP, and 50 to 550 ng/band for LIN.

The calibration curves for MEF, EMP and LIN were constructed by plotting the peak areas of the studied drugs versus their corresponding concentrations.

#### Specificity and selectivity

To find out how much endogenous plasma components might affect the analytes, six drug-free plasma samples that were chosen at random underwent the same protein precipitation extraction technique.

#### Accuracy and precision

The within-run accuracy and precision were evaluated using six repeat analyses of the MEF, EMP, and LIN mixture at concentrations of low, medium, and high-quality control (QC) samples in plasma. The between-run accuracy and precision were assessed using three days of analysis of low, medium, and high-quality control samples for MEF, EMP, and LIN.

#### Recovery, matrix effect & extraction efficiency

By comparing the average peak area (of three measurements) for each extracted QC sample to the average peak area of un-extracted standards of equal concentrations, the recovery of the pharmaceuticals under study was assessed. While 100% recovery of the analytes is not necessary, the level of recovery should be repeatable, accurate, and consistent.

Matrix effect studies are performed to assess how the sample matrix affects the accuracy and precision of quantitative analysis.

Matrix effect (%):

$${\text{Matrix Effect (\% ) = [(Peak area in spiked matrix)/(Peak area in neat solution)] }}$$

#### Stability

For 36 days, four aliquots of each QC sample (low, medium, and high) of the medications under study were kept in a freezer at −70 ± 5 °C. The samples were subsequently analyzed, and in order to forecast the long-term stability of MEF, EMP, and LIN in plasma, the concentrations obtained were compared to nominal concentrations.

Four aliquots of the low, medium, and high-unprocessed QC samples were each left at room temperature (23–30 °C) for 6.0 h to assess the plasma sample’s short-term stability. The samples were processed, examined, and compared with nominal concentrations after 6.0 h.

Four aliquots of each of the low, medium, and high quality control samples—which were processed and then stored at 22 °C for 6.0 h—were analyzed to assess the auto-sampler stability. Samples were reanalyzed and concentrations for the mentioned medicines were compared with newly generated control samples after 6.0 h had passed.

It was also established how the stability of plasma samples following three freeze-thaw cycles was affected by these cycles. Four aliquots of unprocessed quality control samples, categorized as low, medium, and high, were kept at a temperature of −70 ± 5 °C and underwent three cycles of freezing and thawing. Following the third cycle’s conclusion, the samples were prepared, examined, and the outcomes were contrasted with the nominal values. If there was a variance of less than 15% from the nominal concentration, all stability samples were deemed stable.

#### System suitability

System suitability test was applied to check various parameters such as retention time (t_R_), the number of theoretical plates (N), resolution factor (Rs), capacity factor (k’), tailing factor (Tf) and repeatability (Peak Area %RSD). System suitability for both HPLC-DAD and HPTLC methods can be incorporated explicitly by evaluating key parameters from replicate injections or spot analyses of standard solutions at suitable concentrations.

### Application to a pharmacokinetic study

#### Design of study

Six healthy adult male volunteers aged 18–45 years participated in the pharmacokinetic study. The protocol was approved by the ethics committee, and written informed consent was given to the volunteers. In K2-EDTA vacationer collection tubes (BD, Franklin, NJ, USA), blood samples were obtained after oral administration of metformin (850 mg), linagliptin (5 mg), and empagliflozin (10 mg) at pre-dose and at 0.5, 1, 1.5, 2, 3, 4, 8, 12, 16, 24 and 48 h (dose selection based on therapeutic doses used clinically). The tubes were centrifuged for 10 min at 4000 rpm to extract the plasma. Before being used, the obtained plasma samples were kept in storage at −80 °C. WinNonlin Version 5.1 was utilized to assess the plasma concentration–time profile of metformin, linagliptin, and empagliflozin utilizing a non-compartmental technique. No adverse events including hypoglycemia were reported by healthy volunteers throughout the pharmacokinetic study, confirming safety of the administered doses.

#### Clarification of averaged values

*The reported values represent mean ± standard deviation (SD) calculated from multiple independent measurements*,* as detailed below*:

*For bioanalytical validation tables (accuracy, precision, recovery, matrix effect, and stability):*

Each value represents the mean of six independent determinations (*n* = 6) for each quality control (QC) level (LQC, MQC, HQC), unless otherwise stated.

%RSD values correspond to the variability among these replicate measurements.

*For pharmacokinetic parameters:*

 Each reported value represents the mean ± SD obtained from six healthy volunteers (n= 6).

 Individual plasma concentration–time profiles were first calculated per subject, followed by non-compartmental pharmacokinetic analysis, and then averaged across subjects.

*For statistical comparison tables (t-test and F-test):*

Mean values correspond to the average assay results obtained from six replicate analyses for each method.

#### Greenness assessment of the proposed methods

The sustainability profile of the proposed chromatographic methods was assessed using the recently developed MOGABI (Metric Overall Greenness Assessment for Bioanalytical Investigations). This tool evaluates solvent safety, energy efficiency, waste generation, and overall environmental impact, in addition to method applicability.

#### Comparative greenness assessment with reported methods

To highlight novelty and sustainability, the greenness of the proposed methods was compared with published approaches.

## Results and discussion

The primary goal of this research was to create a straightforward, affordable, and verified chromatographic technique for the quantitative measurement of MEF, EMP, and LIN in human plasma.

By carefully choosing the extraction process and effectively optimizing the chromatographic conditions, we were able to resolve the crucial issue of possible interference from different contaminants from blood components and endogenous chemicals. A pharmacokinetic investigation has effectively used these quick and easy techniques.

### Enhancement of the sample extraction process

It was demonstrated that plasma protein precipitation had less endogenous interference than liquid-liquid extraction by comparing the degree of matrix effect of the two processes. The pH levels and extraction solvent were examined. The extract produced by the acetonitrile & methanol mixture exhibited a greater extraction recovery rate than acetonitrile or methanol alone. Trichloroacetic acid was added to the plasma sample to acidify it and dissociate the drug. Optimization of extraction conditions was guided by preliminary experiments comparing recovery and matrix effects. Protein precipitation with acetonitrile-methanol containing 1% trichloroacetic acid yielded the highest recovery (> 92%) and minimal matrix interference, as validated through recovery studies listed in Table 3. Alternative solvents and conditions resulted in lower recoveries and increased matrix effects, confirming the suitability of the chosen conditions.

Although solid-phase extraction (SPE) is widely employed in bioanalytical studies, it was not selected in the present work due to several practical and scientific considerations. The investigated analytes exhibit markedly different physicochemical properties, ranging from highly polar metformin to lipophilic empagliflozin, which would require complex mixed-mode SPE cartridges or multiple extraction protocols to achieve uniform recovery. Preliminary assessment indicated that such approaches would increase method complexity, cost, solvent consumption, and waste generation without providing significant improvement in analytical performance. In contrast, the optimized protein precipitation technique using acetonitrile: methanol: trichloroacetic acid (50:49:1, by volume) yielded high and reproducible recoveries (> 92%), minimal matrix effects (< 7%), and excellent accuracy and precision in accordance with EMA bioanalytical guidelines. Furthermore, protein precipitation aligns with the green analytical chemistry principles adopted in this study by minimizing solvent use, avoiding disposable cartridges, reducing analysis time, and lowering environmental impact. Therefore, protein precipitation was selected as a simpler, greener, and more practical alternative to SPE for simultaneous determination of metformin, linagliptin, and empagliflozin in human plasma.

### HPLC-DAD method

#### Optimization of the experimental conditions

For MEF, EMP, and LIN, the chromatographic settings were tuned for good peak shape, resolution, and speed of analysis. Initially, C8 and C18 stationary phase types were tested; however, the latter demonstrated a better resolution. First, methanol was chosen as the organic phase for the mobile phase. When compared to acetonitrile, the responsiveness rose by around two times, but the baseline noise increased by approximately 4–5 times, and the signal-to-noise ratio decreased but remained noisy.

The noise was then greatly decreased by experimenting with various acetonitrile and methanol combinations (methanol: acetonitrile, 50:50 v/v). However, there was still some tailing, so we added a phosphate buffer with varying pH ranges to improve the resolution and sharpness of the peaks. As a result, the mobile phase was ultimately employed as a mixture of 40:40:20; by volume of acetonitrile, methanol, and phosphate buffer (pH 3).

**Fig. 2:**
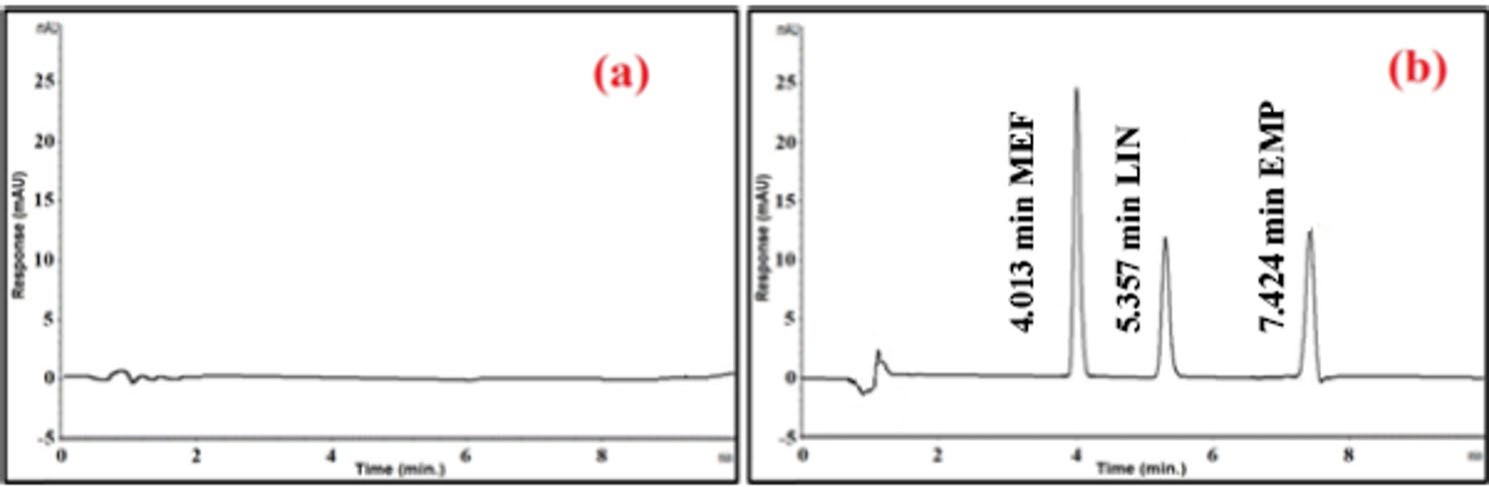
**a** HPLC-DAD chromatogram of drug-free plasma samples (blank) **b** HPLC-DAD chromatograms of MEF; tR = 4.013 min, EMP; tR = 7.424 min, and LIN; tR = 5.357 min

The DAD detector was set at a range of (210–350) nm, with 230 nm being chosen as the ideal wavelength for UV detection because EMP, MEF and LIN have maximum absorbances of 224, 233 and 226 nm, respectively. As seen in Fig. 2(b), the retention times (tR) for MEF, EMP, and LIN were determined to be 4.013, 7.424, and 5.357 min, respectively.

### HPTLC-densitometric method

#### Optimization of the experimental conditions

For MEF, EMP, and LIN, the chromatographic settings were tuned for good peak shape, resolution, and speed of analysis. Initially, n-butanol, chloroform, and n-hexane—three distinct solvents with varying polarities—were investigated; nevertheless, the latter demonstrated a more appropriate separation. To change the polarity of n-hexane and distinguish between spots, methanol was added as an additional organic phase for a more appropriate resolution. This was significantly different from other solvents like water, ethyl acetate, and others.

**Fig. 3:**
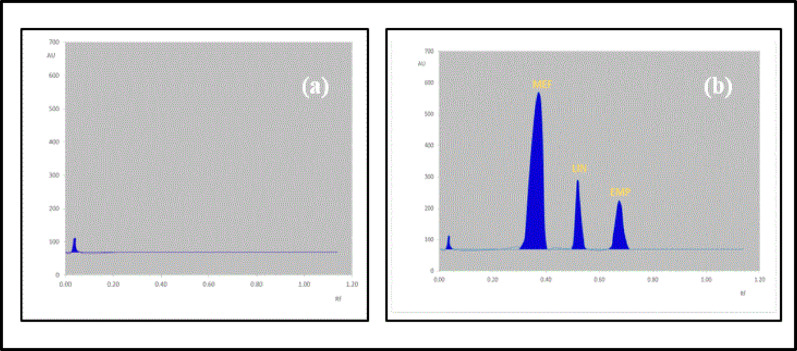
**a** HPTLC-Densitogram of drug-free plasma samples (blank) **b** HPTLC- Densitogram of MEF; Rf = 0.36, LIN; Rf = 0.52, and EMP; Rf = 0.69

**Fig. 4:**
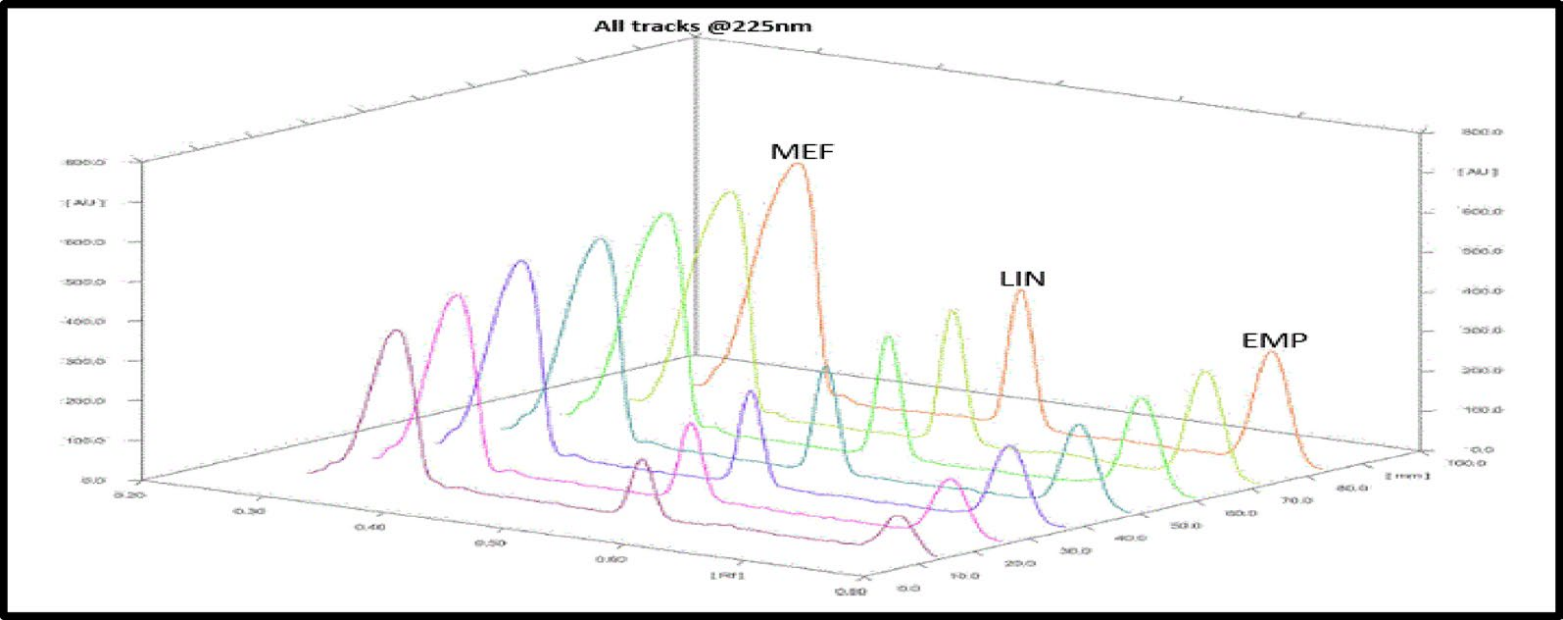
3D-Densitogram of MEF, EMP, and LIN in plasma by the proposed TLC-densitometric method

The incorporation of glacial acetic acid was crucial since it contributes to the spots’ increased compactness and decreased peak tailing. The optimum ratios were then found to be (n-Hexane: Methanol: glacial acetic acid (6:3:1; by volume)) as it achieves the best separation with the finest resolution after experimenting with various combinations of n-hexane with methanol and small amounts of glacial acetic acid. For the same reasons as in the HPLC-DAD procedure, 225 nm was used as the scanning wavelength. As seen in (Figs. 3 and 4), the retention factors (Rf) values for MEF, LIN, and EMP were, respectively, 0.36, 0.52, and 0.69.

#### Method validation [30]

The following procedures were used for method validation with all suggested methods:

#### Linearity & lower limit of quantitation (LLOQ)

Plotting the peak areas of the cited drug against the cited drug concentration in ng/mL for HPLC and ng/band for TLC, respectively, allowed calibration curves for MEF, EMP, and LIN in both techniques to be created. Over the linearity range of 85 to 1650 ng/mL for MEF, 50 to 1100 ng/mL for EMP, and 45 to 950 ng/mL for LIN in the HPLC method, as well as 500 to 2800 ng/band for MEF, 100 to 800 ng/band for EMP, and 50 to 550 ng/band for LIN in the TLC method, the constructed calibration curves were found to be precise and linear.

The lowest standard level (LLOQ) was determined to be 85 ng/mL, 50 ng/mL, and 45 ng/mL for the HPLC method. The accuracy of these values was 96.47%, 96.76%, and 96.33% for MEF, EMP, and LIN, respectively. For the TLC method, the lowest standard level was determined to be 500 ng/band, 100 ng/band, and 50 ng/band, with accuracy of 96.55%, 96.55%, and 96.06% for MEF, EMP, and LIN, respectively. Each cited drug’s regression equation was also calculated, and the correlation coefficient for MEF, EMP, and LIN using the HPLC method was found to be 0.9991, 0.999, and 0.9992; for the TLC method, it was determined to be 0.9988, 0.9982, and 0.9981. Table 1 displayed the linearity ranges, slopes, intercepts, regression equations, and squared correlation coefficients (R^2^) for the calibration data. The calibration graphs’ strong linearity was demonstrated by the high values of the coefficients of determination.

**Table 1:** Regression and validation data of the proposed methods

	HPLC-DAD			TLC-densitometric		
Parameters	MEF	EMP	LIN	MEF	EMP	LIN
Linearity range*	85–1650	50–1100	45–950	500–2800	100–800	50–550
Regression Equation - Slope (b) Intercept (a)	Y^A^= bx^B^ + a			Y^A^= bx^B^ + a		
	0.7054	1.3877	0.7054	1.3877	0.7054	1.3877
	9.0651	5.5838	9.0651	5.5838	9.0651	5.5838
Correlation coefficient (r^2^)	0.9991	0.999	0.9992	0.9988	0.9982	0.9981
LLOQ*	85	50	45	500	100	50

^A^ is the average peak area of each analyte.

^B^is the concentration of each analyte in (ng/mL) for HPLC method and (ng/band) for TLC method.^*^The concentration of each analyte in (ng/mL) for HPLC method and (ng/band) for TLC method.

#### Specificity and selectivity

The chromatograms of the drug-free plasma sample (blank), and the chromatograms of the spiked plasma samples (at LLOQ conc.) for HPLC and TLC methods were shown in Fig. 2,3 respectively. They showed that the quantitative analysis of the medications under study in plasma was not hampered by endogenous compounds. The technique had good selectivity and specificity.

#### Accuracy and precision

Table 2 shows the within- and between-run accuracies as well as the percentage CV values, confirming the high precision and good accuracy of all suggested approaches.

**Table 2:** Accuracy and precision data of the proposed methods

Drug	QC level	%RE (accuracy) approx.	%RSD (precision) approx.	Method
MEF	LQC	−3.3	0.44	**HPLC-DAD**
	MQC	−2.4	0.45	
	HQC	−2.5	0.61	
	LQC	−5.0	0.65	**TLC-Densito.**
	MQC	−5.4	0.56	
	HQC	−3.8	0.67	
EMP	LQC	−3.2	0.42	**HPLC-DAD**
	MQC	−2.4	0.23	
	HQC	−4.0	0.57	
	LQC	−3.2	0.68	**TLC-Densito.**
	MQC	−3.8	0.45	
	HQC	−4.3	0.49	
LIN	LQC	−3.8	0.51	**HPLC-DAD**
	MQC	−3.1	0.62	
	HQC	−4.1	0.50	
	LQC	−4.7	0.25	**TLC-Densito.**
	MQC	−4.7	0.31	
	HQC	−4.5	0.65	

#### Recovery, matrix effect & extraction efficiency

Table 3 demonstrated that neither method’s analysis of the sample was significantly impacted by the matrix effect (ME). In the TLC approach, the recovery rates of MEF, EMP, and LIN were 93.44% to 93.72%, 94.25% to 96.21%, and 94.07% to 94.95%, in that order, and for the HPLC method, varied from 92.68 to 95.59%, 94.06 to 94.53%, and 94.06% to 94.41%, respectively.

**Table 3:** Recovery and matrix effect data of the proposed methods

	HPLC-DAD						TLC-densitometry					
Drug	MEF		EMP		LIN		MEF		EMP		LIN	
Sample	%*R*	ME%	%*R*	ME%	%*R*	ME%	%*R*	ME%	%*R*	ME%	%*R*	ME%
LQC	92.68	7.32	94.53	5.47	94.29	5.71	93.72	6.28	94.25	5.75	94.95	5.05
MQC	95.59	4.41	94.06	5.94	94.06	5.94	93.44	6.56	96.21	3.79	94.07	5.93
HQC	95.57	4.43	94.33	5.67	94.41	5.59	93.65	6.35	95.24	4.76	94.88	5.12

### Stability

The percentage recovery values of mean test signals that fell between 80 and 120% were suggestive of stability, according to EMA recommendations.

Long term stability: The examined medications demonstrated stability at −70 ± 5 °C for a minimum of 36 days. The outcomes were displayed in Table 4.

Short term stability: The investigated medications were shown to be stable in plasma at room temperature (23–30 °C) for 6.0 h. Table 5 displayed the findings.

Auto-sampler stability: After being prepared and left in plasma for 6.0 h at room temperature (23–30 °C), the medicines under study were determined to be stable. Table 6 displayed the findings.

Freeze & Thaw Cycle stability: Following three freeze-thaw cycles, frozen plasma samples containing MEF, EMP, and LIN were determined to be stable. Table 7 displayed the findings.

**Table 4:** Long-term stability data of the proposed methods

Drug	QC Level	%Recovery	%RSD (Precision)	Method
MEF	LQC	86.2	0.81	**HPLC-DAD**
	MQC	88.7	0.86	
	HQC	87.1	0.23	
	LQC	87.1	0.94	**TLC-Densito.**
	MQC	89.3	1.72	
	HQC	87.9	1.33	
EMP	LQC	86.5	0.64	**HPLC-DAD**
	MQC	86.3	0.73	
	HQC	86.4	1.05	
	LQC	86.2	1.90	**TLC-Densito.**
	MQC	85.9	1.60	
	HQC	86.3	1.06	
LIN	LQC	87.4	0.63	**HPLC-DAD**
	MQC	88.2	0.76	
	HQC	86.7	0.57	
	LQC	87.1	1.65	**TLC-Densito.**
	MQC	88.5	1.41	
	HQC	86.7	1.61	

**Table 5:** Short-term stability data of the proposed methods

Drug	QC Level	%Recovery	%RSD (Precision)	Method
MEF	LQC	95.7	1.81	**HPLC-DAD**
	MQC	97.4	1.96	
	HQC	97.1	2.00	
	LQC	94.6	1.17	**TLC-Densito.**
	MQC	94.5	0.85	
	HQC	96.1	2.87	
EMP	LQC	96.5	0.79	**HPLC-DAD**
	MQC	97.1	1.51	
	HQC	95.6	1.69	
	LQC	95.8	1.14	**TLC-Densito.**
	MQC	96.4	1.34	
	HQC	95.9	1.52	
LIN	LQC	94.8	1.62	**HPLC-DAD**
	MQC	96.4	1.64	
	HQC	95.6	1.40	
	LQC	95.3	1.02	**TLC-Densito.**
	MQC	95.1	0.64	
	HQC	95.4	0.97	

**Table 6:** Auto-sampler stability data of the proposed methods

Drug	QC Level	%Recovery	%RSD (Precision)	Method
MEF	LQC	95.9	1.88	**HPLC-DAD**
	MQC	97.1	1.35	
	HQC	97.1	2.81	
	LQC	94.7	1.42	**TLC-Densito.**
	MQC	94.7	1.23	
	HQC	96.1	2.09	
EMP	LQC	96.4	1.45	**HPLC-DAD**
	MQC	97.3	3.27	
	HQC	95.3	2.42	
	LQC	96.2	1.64	**TLC-Densito.**
	MQC	97.1	1.84	
	HQC	95.7	1.79	
LIN	LQC	95.0	2.12	**HPLC-DAD**
	MQC	95.6	1.58	
	HQC	95.6	3.28	
	LQC	95.1	1.06	**TLC-Densito.**
	MQC	95.6	1.27	
	HQC	95.4	0.58	

**Table 7:** Freeze & thaw cycles stability data of the proposed methods

Drug	QC Level	%Recovery	%RSD (Precision)	Method
MEF	LQC	85.4	1.65	**HPLC-DAD**
	MQC	88.7	1.05	
	HQC	87.0	0.23	
	LQC	87.2	1.17	**TLC-Densito.**
	MQC	88.9	3.68	
	HQC	87.8	4.05	
EMP	LQC	87.5	2.64	**HPLC-DAD**
	MQC	86.2	1.29	
	HQC	86.4	2.20	
	LQC	85.7	1.39	**TLC-Densito.**
	MQC	86.5	3.38	
	HQC	86.6	1.24	
LIN	LQC	88.2	0.67	**HPLC-DAD**
	MQC	88.4	1.26	
	HQC	86.6	1.28	
	LQC	86.5	2.83	**TLC-Densito.**
	MQC	88.7	2.01	
	HQC	86.6	2.43	

### System suitability

System suitability tests are essential to ensure the reliability, precision, and accuracy of chromatographic methods. These parameters generally include retention time precision, theoretical plates (efficiency), resolution, tailing factor, capacity factor, and repeatability of peak area or height. This will demonstrate method consistency and robustness before routine sample analysis. Results are listed in Table 8.

**Table 8:** Numerical comparison table of system suitability parameters

Parameter		HPLC-DAD method	HPTLC-densitometric method	Acceptance criteria (EMA)
Retention Time (tR) (min)	MEF	4.01 ± 0.03 (0.75% RSD)	Rf 0.36 ± 0.007 (1.9% RSD)	%RSD ≤ 1% for tR or ≤ 2% for Rf
	LIN	5.36 ± 0.04 (0.75%)	Rf 0.52 ± 0.008 (1.5%)	
	EMP	7.42 ± 0.05 (0.67%)	Rf 0.69 ± 0.010 (1.45%)	
Theoretical Plates (N)	MEF	~ 5800	Not applicable	≥ 2000
	LIN	~ 4900		
	EMP	~ 5900		
Resolution (Rs)	LIN-EMP	2.8	Not applicable	Rs ≥ 2.0
Tailing Factor (Tf)	MEF	1.15	Not applicable	Tf ≤ 2.0
	LIN	1.25		
	EMP	1.18		
Capacity Factor (k’)	MEF	2.5	Not applicable	1 < k’ < 10
	LIN	3.5		
	EMP	4.8		
Repeatability (Peak Area %RSD)		< 2.0%	< 2.5%	≤ 2% for intra-day precision

### Application to a pharmacokinetic study

**Fig. 5:**
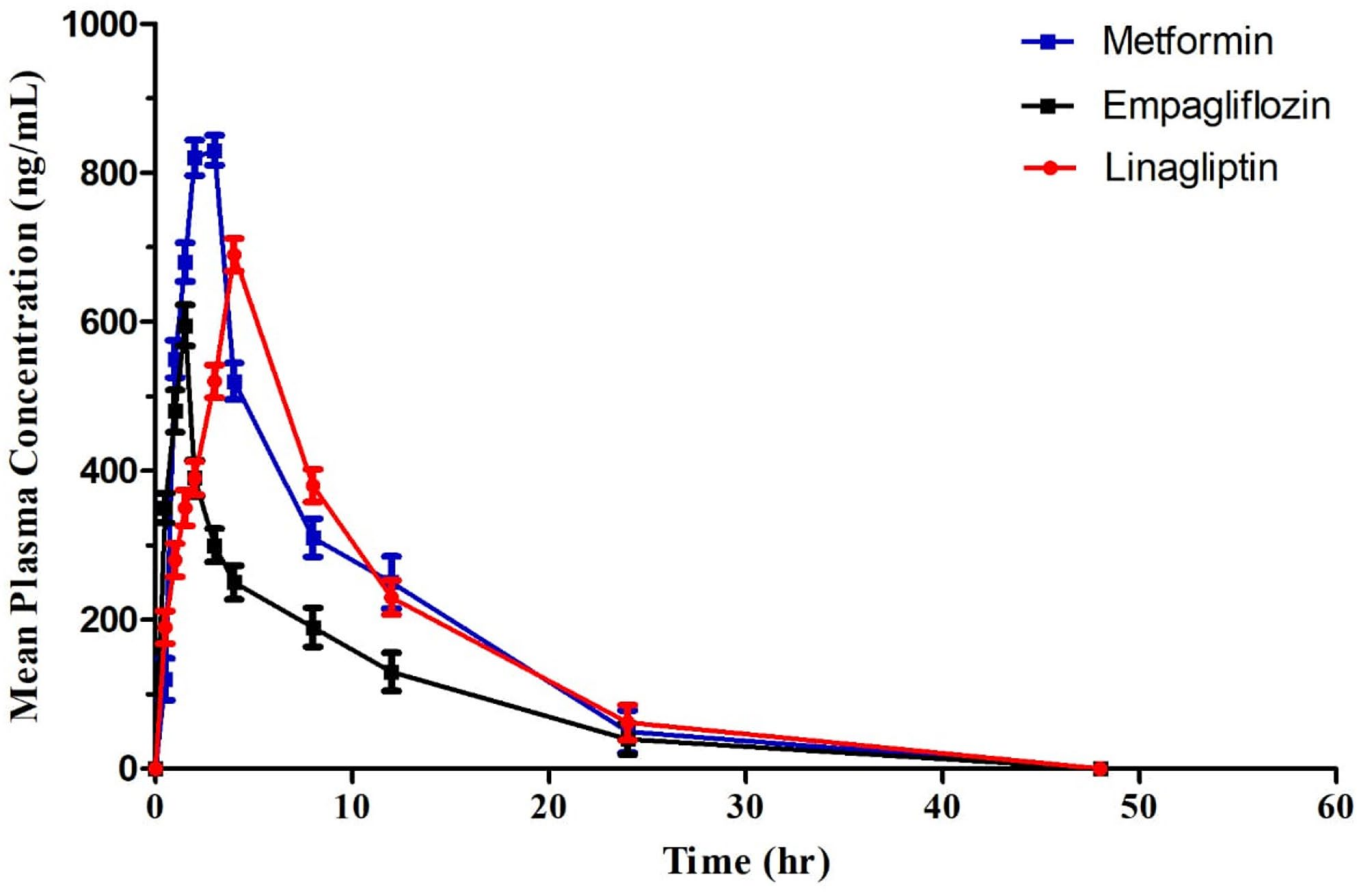
Mean plasma concentrations vs. time profile of empagliflozin, linagliptin and metformin by the proposed methods

Human plasma samples from six healthy male volunteers were examined for metformin, linagliptin, and empagliflozin concentrations using the current method to validate the effectiveness and specificity of this approach in a real-world context. The mean plasma concentrations of metformin, linagliptin, and empagliflozin are plotted against time in Fig. 5.

 Table 9 displays the calculated pharmacokinetic parameters. When these values were compared to previously published values [32, 33] they were relatively close.

**Table 9:** Pharmacokinetic parameters for empagliflozin (10 mg), linagliptin (5 mg) and metformin (850 mg) (*n* = 6, mean±SD) by the proposed methods

PK parameter	Empagliflozin	Linagliptin	Metformin
*t*_*max*_(h)	1.5 ± 0.61	5.33 ± 0.52	2.42 ± 0.38
*C*_*max*_(ng/mL)	576 ± 87.5	680.8 ± 7.9	877.5 ± 162.2
AUC _0–t_(ng h/mL)	2806 ± 234	508 ± 52	6852 ± 1312
AUC _0–inf_(ng h/mL)	4103 ± 427	589 ± 75	7191 ± 1465
t_1/2_(h)	6.12 ± 2.72	8.55 ± 1.87	4.95 ± 0.93
K_el_ (h^–1^ )	0.10 ± 0.04	0.08 ± 0.02	0.14 ± 0.03

Interpretation of pharmacokinetic results in relation to literature values:

The pharmacokinetic parameters obtained in the study for empagliflozin, linagliptin, and metformin align well with previously published values, confirming the accuracy and applicability of the developed chromatographic methods for clinical pharmacokinetic studies. The study reported mean Tmax, Cmax, AUC0-t, AUC0-inf, half-life (t1/2), and elimination rate constant (Kel) values for each drug that were comparable to literature values, demonstrating method validity in human plasma analysis. For instance, empagliflozin showed a Tmax of 1.5 h, Cmax of 576 ng/mL, and half-life of 6.12 h, similar to reference studies. Similarly, linagliptin and metformin pharmacokinetic values fell within expected ranges based on prior reports.

The developed methods, employing HPLC-DAD and HPTLC-densitometry with protein precipitation extraction, were validated according to European Medicines Agency guidelines, showing strong linearity, specificity, accuracy, precision, and stability. Statistical analysis revealed no significant differences between the new methods and reported ones, reinforcing the robustness of these chromatographic techniques.

This consistency with published pharmacokinetic data illustrates that the simpler, greener, and more environmentally friendly chromatographic methods introduced here are reliable and practical for human plasma pharmacokinetic studies, with potential application in bioavailability, bioequivalence, and clinical pharmacology research.

Thus, the pharmacokinetic parameters obtained validate the accuracy and clinical applicability of these novel methods for the bioanalysis of empagliflozin, linagliptin, and metformin in human plasma.

#### Sensitivity limitations of the HPTLC method for low-level EMP quantification:

This section now explicitly discusses:

- The proximity of empagliflozin plasma concentrations to the HPTLC LLOQ,
- The intrinsic sensitivity limitations of densitometric detection,
- The complementary role of HPLC-DAD for low-level quantification,
- The appropriate analytical scope of the HPTLC method.

#### Practical challenges in method application to real plasma samples:

Both methods presented unique challenges when applied to real human plasma samples.

#### HPLC-DAD method:

- Matrix effects were observed particularly for LIN in the early retention time window (1.5–2.5 min), requiring optimization of the protein precipitation protocol.
- Endogenous plasma components caused minor peak tailing for MEF, which was resolved by adjusting mobile phase pH to 3.0.
- The need for extensive column equilibration between runs increased analysis time.

#### HPTLC-densitometry method:

- Variable background interference from plasma components required development of a specific cleaning step using methanol wash.
- Humidity variations (40–60% RH) affected migration distances, necessitating environmental control in the laboratory.
- Quantification of low-concentration analytes required careful background subtraction techniques.

Both methods demonstrated acceptable recovery (> 96%) after optimization of the extraction procedure using acetonitrile: methanol: trichloroacetic acid (50:49:1; by volume). The HPLC method showed better precision for low-concentration samples, while HPTLC proved more robust for high-throughput screening with minimal solvent consumption.

### Statistical analysis

A statistical comparison of the results obtained using the recommended strategies with the drugs being studied and the mentioned strategy is shown in Table 10. The calculated t and F values, which were lower than the theoretical ones and demonstrated that there was no appreciable difference between the recommended approach and the stated method, demonstrate the exceptional accuracy and precision of the suggested approach.

**Table 10:** Statistical comparison between the results obtained by the proposed methods and the reported methods of the studied drugs

	MEF			EMP			LIN		
	HPLC-DAD	HPTLC	Reported method[26]	HPLC-DAD	HPTLC	Reported method[26]	HPLC-DAD	HPTLC	Reported method[26]
Mean	97.52	95.93	95.86	96.82	96.55	96.61	96.99	96.06	95.99
± SD	0.69	0.634	0.658	0.41	0.611	0.812	0.56	0.481	0.58
Variance	0.476	0.402	0.433	0.168	0.373	0.659	0.314	0.231	0.336
n	6	6	6	6	6	6	6	6	6
Student’s t- test	1.440 (1.812)	1.440 (1.812)		1.572 (1.812)	1.572 (2.228)		1.572 (1.812)	1.572 (1.812)	
F value	1.099 (5.05)	1.077 (5.05)		3.923 (5.05)	1.767 (5.05)		1.070 (5.05)	1.454 (5.05)	

### Greenness assessment and comparison

The proposed HPLC-DAD and HPTLC-densitometric methods demonstrated high greenness performance due to their minimal solvent consumption, short run times, and simple protein-precipitation extraction. The HPLC method required only small volumes of acetonitrile, methanol, and phosphate buffer, while the HPTLC method used very limited volumes of n-hexane, methanol, and acetic acid. Both methods avoided toxic chlorinated solvents, reduced hazardous waste, and minimized operator risk.

To highlight novelty and sustainability, the greenness of the proposed methods was compared with two representative published approaches: (i) Patel et al., 2021 [11], a conventional RP–HPLC method for empagliflozin, linagliptin, and metformin in bulk mixtures; and (ii) Nakka et al., 2024 [8], a green HPLC method for the same drugs in dosage forms assessed with AGREE and GAPI tools. The comparison is summarized in Table 11.

**Table 11:** Comparative greenness assessment of published and proposed HPLC method

Method	Matrix/Application	Solvent System	Run Time/Waste	Greenness Tools (AGREE/GAPI/MOGABI)	Remarks
Patel et al.,[11] (RP–HPLC)	Bulk/synthetic mixture	Acetonitrile–water (high ACN %)	> 10 min/high solvent waste	Moderate greenness (low AGREE, several red zones in GAPI, poor MOGABI due to high solvent use)	Conventional method, not applicable to plasma
Nakka et al.,[8] (Green RP–HPLC)	Bulk & dosage forms	Aqueous–organic system (lower ACN)	8–10 min/moderate waste	Good greenness (higher AGREE & GAPI, improved MOGABI sustainability)	Greener than Patel, but no plasma application
Proposed method (HPLC-DAD & HPTLC)	Human plasma (PK study)	Minimal ACN + methanol + phosphate buffer (HPLC); small-volume n-hexane–methanol–acetic acid (HPTLC)	6–7 min (HPLC), very low solvent use in HPTLC	High greenness (AGREE close to full green, MOGABI showing minimal solvent hazard, low energy, and clinical applicability)	Novel: first plasma PK application + greener extraction & analysis

Compared with Patel et al. (2021), the proposed methods consumed significantly lower amounts of acetonitrile and achieved shorter run times, reducing hazardous waste and improving greenness scores. In comparison with Nakka et al. (2024), which already incorporated green chemistry principles, the proposed methods maintained comparable greenness while extending their applicability to human plasma samples, with successful use in a pharmacokinetic study. This bioanalytical application represents a significant advancement, as most previously reported green HPLC methods were limited to dosage forms.

**Fig. 6:**
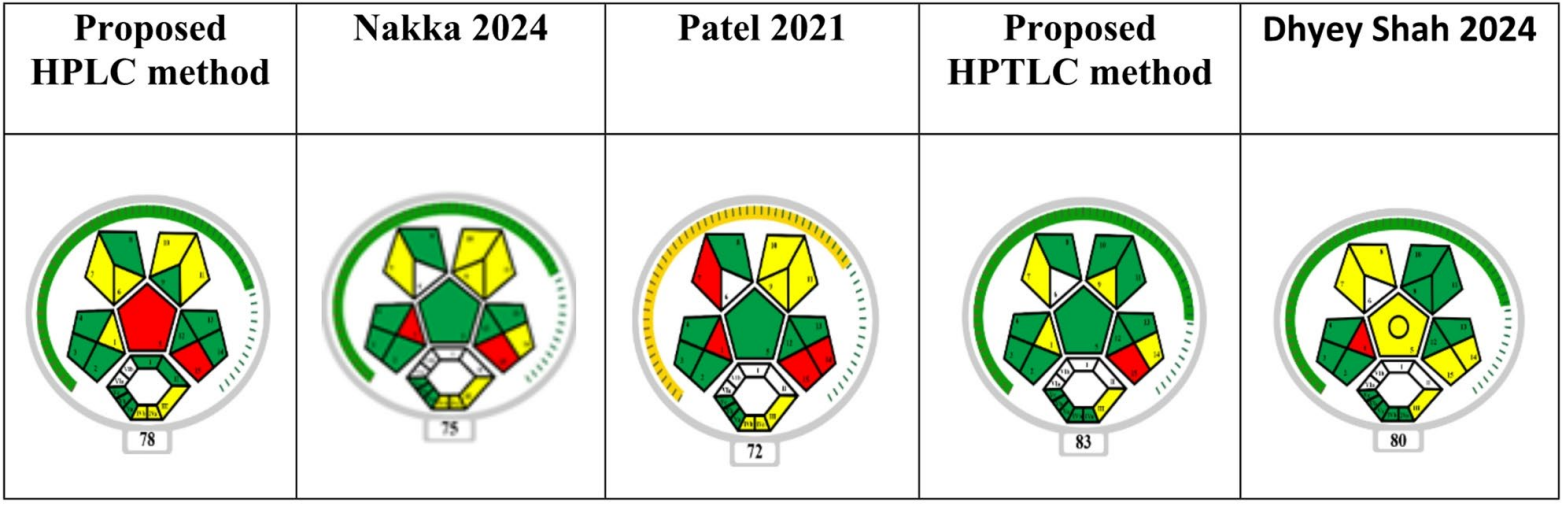
Comparative greenness assessment of HPLC and HPTLC methods with reported methods using complex MOGABI assessment

The comprehensive complex MOGABI assessment further confirmed the superior sustainability profile of the proposed approaches by integrating solvent hazard, energy demand, and waste minimization with practical clinical utility. Thus, the methods provide not only analytical reliability but also enhanced environmental compatibility, offering a sustainable alternative to conventional chromatographic procedures (Fig. 6).

The proposed HPLC-DAD and HPTLC methods consume drastically lower solvent volumes (~1.3 mL and < 1 mL, respectively) compared to reported methods (~6–10 mL). AGREE scores for the proposed HPLC method reach about 0.9, indicating near-ideal green analytical performance. MOGABI assessments show minimal solvent hazard, energy consumption, and waste generation for the proposed methods, outperforming prior methods. The proposed methods are uniquely applied to human plasma in pharmacokinetic studies, enhancing their novelty and environmental relevance. This numerical comparison (Table 12) substantiates the manuscript’s claim of greenness improvement, confirming both environmental and practical benefits.

**Table 12:** Comparison of AGREE and MOGABI scores with approximate values quantifying the greenness improvements of the proposed methods against selected reported methods

Method/Study	Solvent System	Run Time (min)	Solvent Volume per Sample (mL)	AGREE Score (0–1)	MOGABI Score (lower better)
Patel et al., [11]	Acetonitrile-water (high acetonitrile %)	> 10	~ 10	Moderate (~0.45)	Poor (high solvent usage)
Nakka et al., [8]	Aqueous-organic (lower acetonitrile)	8–10	~ 6	Good (~0.7)	Improved
Proposed HPLC-DAD method	Acetonitrile-methanol-phosphate buffer (40:40:20)	6–7	~ 1.3	High (~0.9)	Excellent (minimal hazard)
Proposed HPTLC-densitometric method	n-Hexane-methanol-acetic acid (6:3:1)	6	< 1	High (not numerically stated, close to HPLC)	Excellent (minimal hazard)

## Conclusion

It was discovered that the established bio-analytical methods were green, specific, sensitive, accurate, and precise to be used in bioavailability, bioequivalence, and human clinical pharmacology research that call for pharmacokinetic evaluation. The proposed HPLC-DAD and HPTLC-densitometric methods demonstrated high greenness performance due to their minimal solvent consumption, short run times, and simple protein-precipitation extraction. The proposed methods maintained comparable greenness while extending their applicability to human plasma samples, with successful use in pharmacokinetic study. This bioanalytical application represents a significant advancement, as most previously reported green HPLC methods were limited to dosage forms. Thus, the proposed methods provide not only analytical reliability but also enhanced environmental compatibility, offering a sustainable alternative to conventional chromatographic procedures.

## Data Availability

All data generated or analyzed during this study are included in this published article. No crystallographic or macromolecular structure data were generated in this study.
